# Microstructural examination of carbonated 3D‐printed concrete

**DOI:** 10.1111/jmi.13087

**Published:** 2022-02-20

**Authors:** Asel Maria Aguilar Sanchez, Timothy Wangler, Matteo Stefanoni, Ueli Angst

**Affiliations:** ^1^ Physical Chemistry of Building Materials Institute for Building Materials, ETH Zurich Zurich Switzerland; ^2^ Durability of Engineering Materials Institute for Building Materials, ETH Zurich Zurich Switzerland

**Keywords:** carbonation, concrete, digital fabrication, 3D printing, lubrication layer, microstructure

## Abstract

The recent interest in 3D printing with concrete has generated great interest on how inhomogeneities arise and affect performance parameters, in particular strength and durability. With respect to durability, of particular interest is how 3D‐printed layer interfaces can impact transport of species of interest, such as moisture, chlorides or carbon dioxide in carbonation processes. This is of particular interest considering that the primary use case of 3D‐printed concrete has been as a lost formwork for a cast structural concrete, and thus it is of interest to determine the carbonation resistance. This study consists of a preliminary look at the microstructure after accelerated carbonation of a 3D‐printed concrete used as a lost formwork. Preferential carbonation is observed in the layer interfaces compared to the bulk of the printed filaments, possibly related to porosity from air voids or a locally high capillary porosity corresponding to the lubrication layer.

## Lay Description

The new technology of 3D printing with concrete has been making a lot of headlines recently due to its great potential to make construction safer, cheaper and faster. It also allows us to make buildings and infrastructure objects that are more materially efficient, meaning that they use much less concrete compared to a more standard construction, so they are less environmentally harmful. However, this is all assuming that the printed concrete will perform similar to normal concrete. A lot of attention has been paid to whether the printed concrete is as strong as normal concrete, however not so much attention has been paid to if the printed concrete is as durable as normal concrete. The aim of this study is to make a first look at this, using the microscope.

When we speak of concrete durability, we typically mean the protection of the steel reinforcement in the concrete, which acts to take up tensile stresses that may arise. Concrete acts as a protective barrier to this steel from corrosion, but aggressive species can go through this barrier to attack the steel. One of these aggressive species is carbon dioxide, which acts to reduce the pH around the reinforcement and results in its corrosion. Printed concrete, made in a layer‐by‐layer process, has many interfaces between these layers where the connection is potentially not as dense as in normal concrete. This study shows that these layer interfaces essentially can serve as highways for carbon dioxide to enter the concrete and attack the reinforcement. This means that any new 3D‐printed structures need to take this into account, if the printed concrete is expected to serve as any kind of a protective barrier. We caution the reader that this study is purely observational, however, and a more in‐depth study where we can actually make predictions about the printed concrete should be carried out.

## 1 INTRODUCTION

Digital fabrication with cementitious materials, or digital fabrication with concrete (DFC) has emerged in recent years as a hot topic in research due to its potential to revolutionise a long‐stagnant construction sector.^1^, ^2^ Numerous benefits are hoped to be realised with the technologies, particularly increased productivity and increased sustainability of concrete components. These two benefits are expected to be derived primarily from the hallmark of the technology, which is the removal of traditional formwork: more shape freedom for more material‐efficient structures enhances sustainability, and removal of formwork labour removes a major labour cost and time burden in production of concrete components.^3^

The dominant DFC method employed to date has been layered extrusion^4^, ^5^ (often called 3D concrete printing, or Contour Crafting, the brand name of its first major developer). The method consists of subsequent placement of filaments of cementitious material to eventually build up a component. The method has until now primarily been utilised to produce lost formworks, because of the inability to efficiently incorporate traditional steel reinforcement in the vertical direction.^6^, ^7^, ^8^ In fact, the most visible industrial use case has been the production of masonry structures with integrated formworks for structural columns, to which passive reinforcement is later added and a structural concrete cast.^3^, ^9^, ^10^, ^11^, ^12^ This method inherently produces a layered structure, and while the effect of the layer interfaces has produced most research interest related to their effect on the mechanical properties,^13^, ^14^, ^15^, ^16^ recently research has started to focus on how the layer interfaces can impact durability.^17^ To that end, some studies have focused on moisture transport in the layer interfaces,^18^, ^19^ and some recent studies have focused on chloride transport as well.^20^, ^21^ Other studies have explicitly focused on analysis of the microstructure of the layer interfaces, with the air void system and the porosity being the primary focus point.^22^, ^23^, ^24^, ^25^, ^26^ Until now, however, only one very recent study has examined carbonation of printed concrete,^27^ and in this study, the microstructure of the carbonated concrete was not examined. Additionally, until now, most studies have focused on unaccelerated 3D‐printed mixes, which generally have high viscosities, high initial yield stresses, and low buildability rates.

In this study, we report results on the microstructure of carbonated printed concrete as examined by optical thin section and SEM‐EDX. The concrete of this study is also printed through use of a set accelerator, with a low viscosity and low initial yield stress but a rapid buildability rate. Samples were produced as they would be expected to be used, that is through the use of 3D‐printed concrete as a formwork for a cast concrete. Good structural engineering design would demand the placement of reinforcement bars as close to the edge of the structural element as possible (where tension is highest), but design codes also demand a concrete cover to mitigate corrosion by carbonation. Therefore, it is worth examining if printed concrete could conceivably be used as a cover layer in 3D‐printed components, and our analysis of this 3D‐printed sample is made with this in mind.

## 2 MATERIALS AND METHODS

### 2.1 Material mixes

#### 2.1.1 3D‐printed mix

The mix used for 3D printing has also been reported elsewhere.^8^, ^28^ Briefly, it consists of a CEM I binder (Holcim Normo 5) with fine limestone and silica fume substitutions (15% and 8%, respectively), a superplasticizer, a viscosity modifier and sucrose to ensure the mix open time. This is added to a crushed limestone sand with max grain size of 2 mm, mixed with water at *w*/*b* of 0.39 and sand to binder ratio of 1.9, before eventually being mixed in the print nozzle with an accelerator paste consisting of calcium aluminate cement (CAC) which is also retarded with sodium gluconate, at an approximate dosage of 10% OPC substitution.

#### 2.1.2 Cast concrete mix

The mix for the cast concrete was a standard C20/25 mix with a CEM I 42.5 N (Holcim Normo 4) at *w*/*c* = 0.6 and maximum grain size of 16 mm.

### 2.2 Sample production and carbonation

Printed samples were produced by the 3D printing process at ETH Zurich also described in.^8^, ^28^ They were first printed as hollow square (25 cm × 25 cm) specimens with an approximate filament width of 3.5–4 cm and a layer height of 0.5 cm, up to a total height of 25 cm. The robot moved at a speed of 200 mm/s, thus with an effective layer interval time of a few seconds. After printing, the samples were cured in ambient conditions for 10 days before the cast concrete was added within the printed formwork. The concrete was stored at ambient conditions for another 2 months before they were placed in a carbonation chamber.

The samples were carbonated in accelerated conditions in a self‐made carbonation chamber made of a sealed box connected to a CO_2_ gas bottle. The CO_2_ level was maintained between 50% and 90% at a RH of 70% for a period of 2 months before the samples were removed.

### 2.3 Sample preparation for microscopy

The produced samples were then cut and immediately had phenolphthalein and thymolpthalein applied to check for carbonation depth. Regions of interest were identified and a polished thin section was produced from layers approximately middle height (as there was no major variation observed in carbonation behaviour from top to bottom) to observe the microstructure with both optical and scanning electron microscopes.

### 2.4 Microscopy

A stereo microscope Leica M 60 was used for the observation of bulk samples and a Leica DM750 P in both plane and polarised light mode was used for observing the thin section. SEM‐EDX observation (BSE images) and point analyses were performed with a QUANTA 2003D and EDX system from EDAX company.

## 3 RESULTS

**FIGURE 1:**
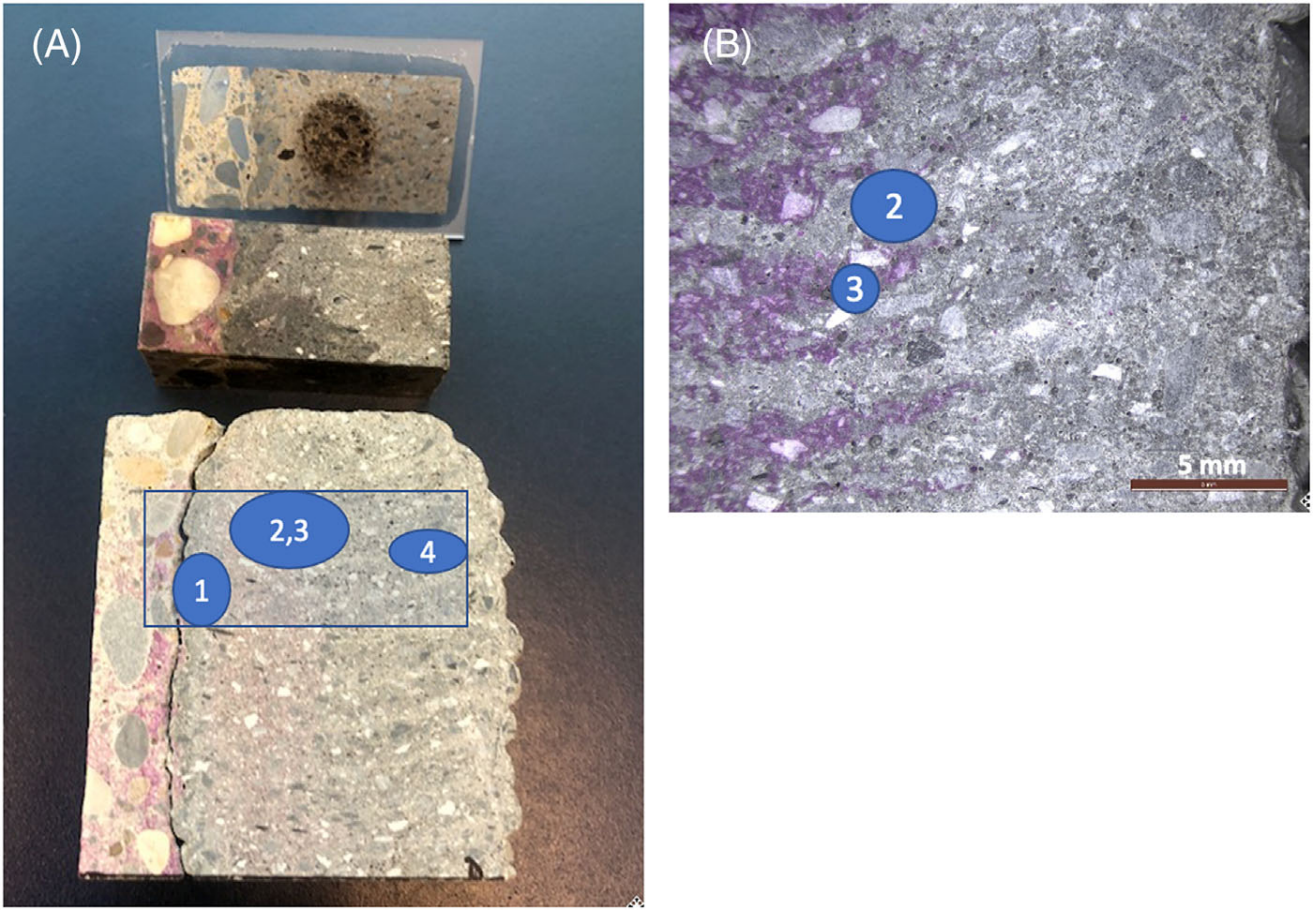
sample and corresponding thin section area (A), with zones labelled. Zone 1: close to cast–print interface. Zone 2, 3 (B): within ‘transition zone’ containing uncarbonated concrete (purple colour from phenolpthalein indicates uncarbonated zones), with 2 focused on the carbonated zone in the layer interface, and 3 focused on the uncarbonated zone. Zone 4: fully carbonated zone

Figure 1 shows the sample from which the thin section was taken, with zones of interest labelled. It can be seen that significant carbonation has taken place over the depth of the printed concrete, and that this occurs inhomogeneously, apparently corresponding to a higher degree of carbonation in the layer interfaces than in the bulk of the interlayers. There exist four interesting zones in the printed sample. First, there is a zone next to the cast concrete that is carbonated next to an uncarbonated zone (Zone 1), then there is a ‘transition zone’ consisting of carbonated concrete in the interlayer zone and uncarbonated concrete in within the bulk of the layer (Zones 2 and 3, respectively) and then there is a fully carbonated zone extending from the exposure surface to approximately 2–3 cm into the printed concrete (Zone 4).

**FIGURE 2:**
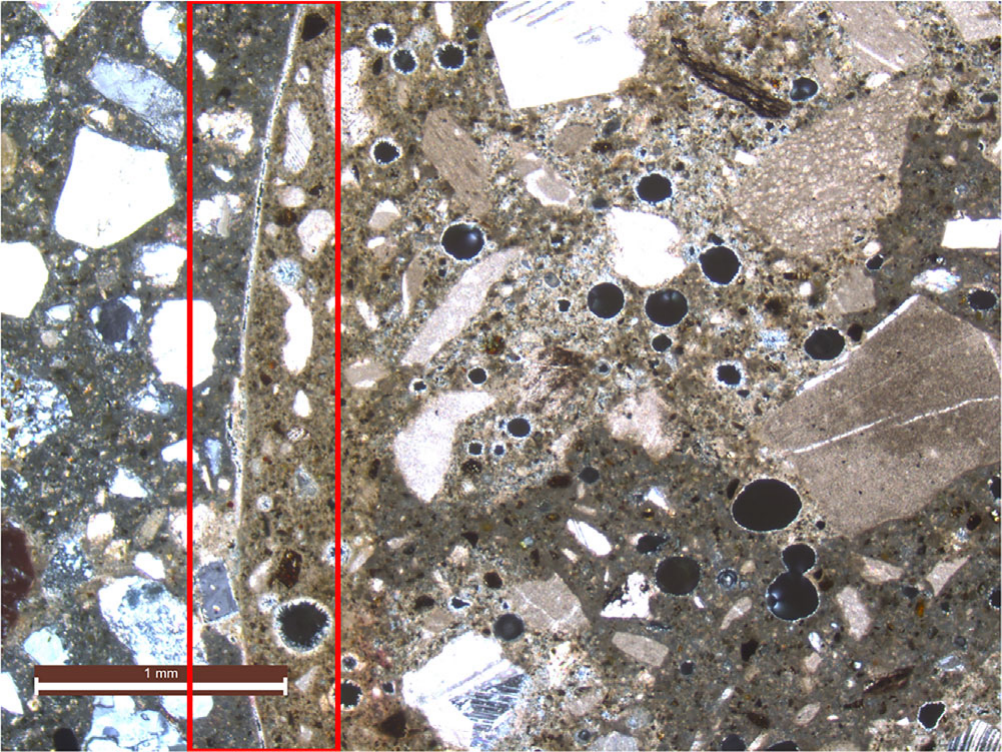
Cross polarized micrograph of Zone 1 (cast–print interface, cast concrete on left). Carbonation front observed (red box) and precipitation of calcite crystals in air voids and interfacial porosity

In Figure 2, one sees an optical thin section of the cast–print interface. One can clearly see here a uniform and very dense carbonation front extending to approximately 0.5 mm from the cast–print interface, and the precipitation of calcite crystals (confirmed by SEM‐EDX) in air voids and within the interface. It should also be noted that in SEM‐BSE, it was observed that the degree of hydration in this zone was quite advanced, with almost no anhydrous cement observed. It was also observed that carbonation was not homogeneous, with very dense areas and other areas of high porosity. General microcracking was observed everywhere within the printed concrete as well.

**FIGURE 3:**
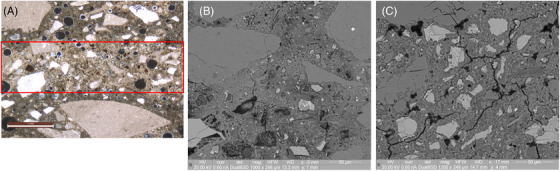
cross‐polarized optical micrograph of Zones 2 and 3 (A) and SEM‐BSE micrograph of Zone 2 (B) and Zone 3 (C). Clearly carbonated region seen approximately 1 mm in thickness (in red) at the printed layer interface. Larger unhydrated cement particles apparent in Zone 3 compared to Zone 2

Figure 3 focuses on Zones 2 and 3, where a cross‐polarised optical micrograph shows a clearly densified region of carbonation approximately 1 mm in thickness at the layer interface. SEM‐BSE images focused on each of the zones show a difference in hydration degree, with the bulk layer region (Zone 3) tending to show lower degree of hydration, with larger unhydrated particles. In the carbonated region, similar to Zone 1, the carbonation was not homogeneous. Microcracking was also observed everywhere.

**FIGURE 4:**
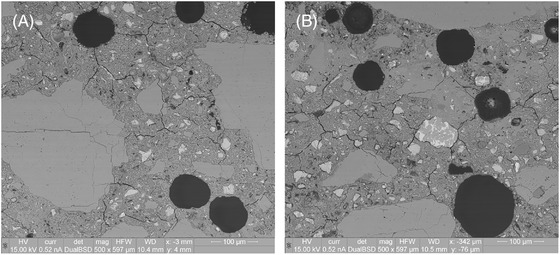
SEM‐BSE images of fully carbonated region of Zone 4, with interfacial zone (A) and bulk zone (B). Higher degree of hydration observed in interfacial zone compared to bulk

Figure 4 shows SEM‐BSE micrographs of Zone 4, focusing on both the interfacial and the bulk layer zones as well. Similar to Figure 3, larger unhydrated cement particles with more poorly developed hydration rims are seen in the bulk region compared to the layer interface region. The amount of carbonation observed is also still higher in the layer interface region compared to the bulk region, and again microcracking is observed everywhere. Finally, it was observed that the air void sizes tended to be larger at the layer interface region.

## 4 DISCUSSION

At the cast–print interface, one can observe a separation between the printed and cast layers, which could be due to drying shrinkage differences between the two materials and their different production times. The uniform carbonation depth of 0.5 mm in the printed concrete is likely due to the 10 days delay between the printing and the casting, however, because no corresponding carbonation depth was observed in the cast concrete. It is also worth noting that the highly advanced degree of hydration in this zone could be due to sorption of water from the cast concrete. Overall, examination of this region highlights the importance of understanding the relationship between printed formworks and cast concrete in terms of the overall process and performance.

In the ‘transition zone’ (Zones 2 and 3), it is quite apparent that carbonation is occurring nonuniformly within the printed concrete, and that it is occurring with greater speed within the layer interfaces, indicative of a difference in the diffusivity between the layer interfaces and the bulk. This higher carbonation speed in the layer interfaces contributes to faster carbonation in the bulk of the print filament as well, essentially by allowing carbonation from the top and bottom of the filament and effectively reducing the diffusion length.

It is interesting to note that the thickness of the carbonation layer at the layer interface is approximately 1 mm. It is well known that a ‘lubrication layer’ forms during pumping of concrete, in which a paste rich zone forms at the wall of the pipe,^29^, ^30^ and it has been noted that this lubrication layer could also be present after extrusion of concrete, and thus responsible at least in part for heterogeneity across the filament cross section.^31^ The size of this lubrication layer has been measured through various techniques and estimated to be on the order of 1–5 mm.^32^ This increase in paste content, however, is likely not responsible for the enhanced transport in the layer interfaces, because it has been demonstrated that paste volume does not play a large role in the carbonation depth.^33^ It is much more likely that there is another factor at play, and the most likely reasons are increased porosity from air voids, or increased capillary porosity.

As noted earlier, most studies to date have focused on presence of defects and air voids in the interlayer, with a primary focus on their effect on the strength. Numerous X‐ray micro computerised tomography studies have detailed their presence and extent with respect to processing parameters such as printhead speed, pressure, and height, layer interval time, and ambient conditions with respect to moisture,^23^, ^24^, ^25^, ^26^, ^27^ with only one directly linking the measurements to durability performance metrics.^27^ However, the rheology of each of these systems stands in stark contrast to the rheology of the 3D printing system used in this study. The rheology in this study is much more fluid, practically self‐compacting upon exit from the nozzle (yield stress ∼500 Pa, with a correspondingly low viscosity^34^), and thus fluid enough to allow entrapped air to escape. While it was observed that the air voids were slightly larger in the layer interfaces, other possibilities for faster carbonation should be explored, including the possibility of increased capillary porosity.

Capillary porosity is strongly influenced by the *w*/*c* ratio. The same physical principle that leads to a depletion of aggregate next to the wall will also lead to a depletion of binder particles, which would lead to an effective increase in the *w*/*c* ratio within a certain range of the layer interface. This moisture content is enhanced by pressurised bleeding, noted by Sanjayan et al.^15^ and Kosson et al.^35^ A locally higher *w*/*c* ratio would manifest itself in a higher degree of hydration, which was observed in this sample, across the width of the layer interface. This is corroborated by Van Der Putten et al.,^26^ who also found a lower proportion of unhydrated cement particles at the layer interface. This phenomenon could be the source of the higher degree of hydration at the cast–print interface, as the lubrication layer is formed around the entire extrusion filament. In any case, a locally high *w*/*c* at the layer interface would lead to a higher capillary porosity and thus enhanced transport.

The dense carbonation layer at the layer interface could also be indirect evidence of this locally high capillary porosity. One would expect that carbonation products would form generally where there is a high amount of portlandite, such as in the traditional interfacial transition zone (ITZ). For example, Shen et al. demonstrated that carbonation products formed significantly in the ITZ.^36^ The layer interface could also be considered similar to an ITZ, and a locally high *w*/*c* and concomitantly higher capillary porosity would allow portlandite to precipitate there preferentially, thus also to carbonate there preferentially.

A final discussion point could be made regarding the high degree of microcracking observed. While this could easily be related to sample preparation, it could also bear some relation to material, processing, and shrinkage. The printed material is essentially a binary OPC‐CAC system, relying on ettringite formation to develop rapid buildability strength.^37^ Inhomogeneities in mixing and especially across the filament cross section could lead to differential displacements due to differential shrinkages from drying, or from carbonation of the portlandite or ettringite. The microcracking, if it comes from one of these processes, could also act as an enhancer of CO_2_ transport.

## 5 CONCLUSIONS AND OUTLOOK

The primary conclusions and recommendations that can be drawn from this preliminary look at the microstructure of carbonated 3D‐printed concrete are the following:

- The 3D‐printed concrete of this study showed nonuniform carbonation due to the presence of the layer interfaces, with the layer interfaces showing faster carbonation. This can be from enhanced transport due to either increased air content or capillary porosity in the layer interface. The implications for this require further study, as this would negatively impact the possibility of using a printed formwork as a cover layer.
- The interface of a structural concrete cast into a 3D‐printed formwork (‘cast–print’ interface) requires attention in future studies, especially as this represents the predominant use case of this technology.
- A more complete study is needed regarding the generation and evolution of microstructural heterogeneities in 3D‐printed concrete with respect to carbonation, in particular with respect to the capillary porosity at the layer interfaces.
